# Proteomic analysis of serum small extracellular vesicles identifies diagnostic biomarkers for neuroblastoma

**DOI:** 10.3389/fonc.2024.1367159

**Published:** 2024-08-20

**Authors:** Juan Cheng, Dongrui Ji, Jing Ma, Qinghua Zhang, Wanglin Zhang, Lin Yang

**Affiliations:** ^1^ Department of Clinical Laboratory, Shanghai Children’s Medical Center, School of Medicine, Shanghai Jiao Tong University, Shanghai, China; ^2^ Wayen Biotechnologies (Shanghai), Inc., Shanghai, China; ^3^ Department of Pathology, Shanghai Children’s Medical Center, School of Medicine, Shanghai Jiao Tong University, Shanghai, China; ^4^ Department of Orthopaedics, Shanghai Children’s Medical Center, School of Medicine, Shanghai Jiao Tong University, Shanghai, China

**Keywords:** neuroblastoma, extracellular vesicles, proteomics, biomarker, pediatrics

## Abstract

**Background:**

Neuroblastoma (NB) primarily arises in children who are <10 years of age, and originates from developing sympathetic nervous system, which results in tumors in adrenal glands and/or sympathetic ganglia. The diagnosis of NB involves a combination of laboratory and imaging tests, and biopsies. Small extracellular vesicles (sEVs) have gained attention as potential biomarkers for various types of tumors. Here, we performed proteomic analysis of serum sEVs and identified potential biomarkers for NB.

**Methods:**

Label-free proteomics of serum sEVs were performed in the discovery phase. A bulk RNA-seq dataset of NB tissues was used to analyze the association between genes encoding sEVs proteins and prognosis. Potential biomarkers were validated via multiple reaction monitoring (MRM) or western blot analysis in the validation phase. A public single-cell RNA-seq (scRNA-seq) dataset was integrated to analyze the tissue origin of sEVs harboring biomarkers.

**Results:**

A total of 104 differentially expressed proteins were identified in NB patients with label-free proteomics, and 26 potential biomarkers were validated with MRM analysis. Seven proteins BSG, HSP90AB1, SLC44A1, CHGA, ATP6V0A1, ITGAL and SELL showed the strong ability to distinguish NB patients from healthy controls and non-NB patients as well. Integrated analysis of scRNA-seq and sEVs proteomics revealed that these sEVs-derived biomarkers originated from different cell populations in tumor tissues.

**Conclusion:**

sEVs-based biomarkers may aid the molecular diagnosis of NB, representing an innovative strategy to improve NB detection and management.

## 1 Introduction

NB is the most common solid tumor in early childhood that originates from precursor cells of the sympathetic nervous system, and accounts for up to 10% of childhood cancer deaths (1). Despite intensive treatments, the outcome for patients with high-risk NB still remains poor (2–4). Although advancements in molecular and genetic testing of tumor tissues have improved the accuracy and precision of NB diagnosis (5–7), biopsies or bone marrow aspirations are invasive and uncomfortable for patients, especially young children. Therefore, identification of more convenient biomarkers is needed to further improve the accuracy, early detection, or risk stratification of NB patients.

Small extracellular vesicles (sEVs) are secreted by different living cells and carry characteristic substances from their parental cells. In particular, increasing evidence has shown that sEVs have the ability to carry tumor-specific molecules that reflect the genetic and molecular characteristics of tumor cells (8–11), promoting the research progress of sEVs as tumor biomarkers. In addition, sEVs offer several advantages as biomarkers, for example, sEVs can be collected noninvasively, the lipid bilayer of sEVs protects cargo from degradation, and the isolation and analysis of sEVs can be performed using relatively standard laboratory techniques, making them accessible for research and clinical applications (12).

Recently, proteins or RNAs carried by sEVs have been shown to serve as candidate biomarkers for various types of tumors, such as cholangiocarcinoma (11), colorectal cancer (13), ovarian cancer (14), prostate cancer (15), pancreatic ductal adenocarcinoma (16), esophageal cancer (17), and gastric cancer (18, 19). In terms of clinical transformation, it is worth mentioning that the ExoDx Prostate test, based on the expression analysis of three tumor-related genes in urine sEVs, has been used in clinical practice for early detection of prostate cancer (20).

Furthermore, given the ability of sEVs to harbor tumorigenicity, inhibiting sEVs formation or release may be a strategy for treating tumors (21–24). In addition, immune cell-derived sEVs such as dendritic cell sEVs (25, 26), have also potential for cancer immunotherapy. sEVs are also ideal candidates for drug delivery due to their biocompatibility, stability, and targeting capabilities (27).

Taken together, these findings indicate that sEVs hold promise as potential sources of tumor biomarkers as well as targets and tools for cancer therapy. Herein, we performed proteomic analysis of serum sEVs, and identified potential biomarkers for the diagnosis of NB.

## 2 Materials and methods

### 2.1 Patients

A total of 30 NB patients, 30 HC and 10 non-NB patients, including those with pilomatricoma (n=4), teratoma (n=2), mucinous cystadenoma (n=1), Hodgkin lymphoma (n=1), hepatoblastoma (n=1) and an unknown tumor (n=1), between October 2020 and January 2022 were enrolled in this study. Among these participants, 10 NB patients and 10 HC were included in the discovery phase, and the remaining were included in the validation phase. The clinical characteristics of the patients are shown in **Supplementary Table 1**. Tumor tissues were obtained, stained with hematoxylin and eosin (H&E) and examined by an experienced pathologist. Histological characteristics were evaluated.

### 2.2 Isolation and characterization of serum sEVs

Peripheral blood samples were collected and then immediately processed to isolate serum using BD Vacutainer SST Serum Separation Tubes (Becton Dickinson, Oxford, England). The collected serum samples were routinely stored at −80°C until used. This study was a retrospective study. Serum samples collected between October 2020 and January 2022 before the start of the study were used for analysis. 1ml of frozen serum samples were thawed and then centrifuged. The supernatant was subjected to sEVs extraction using qEV size exclusion columns (qEVoriginal/70 nm Legacy, IZON Science, Christchurch, New Zealand) as described previously (28). sEVs were collected and then concentrated to 250 µL using an Amicon^®^ Ultra 10K centrifugal filter (Millipore, Burlington, MA). The particle and protein concentrations were determined via nanoparticle tracking analysis (NTA) using a ZetaView^®^ nanoparticle tracking analyzer (8.05.05 SP2; Particle Metrix GmbH, Meerbusch, Germany) and bicinchoninic acid (BCA) assay, respectively. The ultrastructure of the sEVs was analyzed via transmission electron microscopy (TEM) (Tecnai G2 Spirit BioTwin, FEI, Oregon).

### 2.3 Western blot

sEVs proteins were extracted using RIPA lysis buffer (Beyotime, China). Briefly, an appropriate volume of RIPA was added to the PBS solution containing sEVs (1:1), and the mixture was incubated at 4°C for 30 min, followed by centrifugation at 12000g at 4°C for 15 min. The supernatants containing sEVs proteins were collected. Protein concentrations were determined by the Pierce™ BCA Protein Assay Kit (Thermo Scientific, Waltham, MA) according to manufacturer’s instructions. 10μg of proteins were separated using the 10% SDS-PAGE gel. Following electrophoresis, the gel was transferred onto a PVDF membrane. After blocking with 5% skimmed milk at room temperature for 60 min, the membrane was then incubated overnight at 4°C with following primary antibodies: anti-CD9 (1:1000, SBI, CA), anti-Hsp70 (1:1000, Abcam, Cambridge, UK), anti-HSP90AB1 (1:500, Immunoway, TX), anti-SLC44A1 (1:500, Immunoway, TX), anti-CHGA (1:500, Immunoway, TX), anti-BSG(1:500, Immunoway, TX), and anti-ITGAL (1:500, Immunoway, TX). After incubation with the secondary antibody, the membrane was incubated a super ECL detection reagent (Yeasen, China), and then detected on an automatic chemiluminescence image analysis system (Tanon 5200 Multi, China).

### 2.4 Liquid chromatography−tandem mass spectrometry

Purified sEVs were lysed in buffer containing 7 M urea, 2% sodium dodecyl sulphate (SDS), and 1× protease inhibitor cocktail and digested using the standard filter aided proteome preparation protocol as described previously (29). Briefly, for each sample, 50μg of proteins were reduced with 20 mM dithiothreitol at 57°C for 60 min, alkylated with 90 mM iodoacetamide for 40 min, and digested with trypsin overnight at 37°C. After centrifugation and concentration, the peptides were desalted by MonoSpin column (5010-21701, GL Sciences, Tokyo, Japan). The desalted peptides were centrifuged and dried.

LC−MS/MS was performed with a QE-HF-X mass spectrometer coupled to an EASY-nLC 1200 (Thermo Scientific, Waltham, MA). The desalted peptides were reconstituted with 0.1% formic acid (FA), transferred to an analytical column (C18, 2 µm, 100 Å, 50 µm × 15 cm; Thermo Scientific, Waltham, MA), and separated using an EASY-nLC 1200 at a flow rate of 300 nL/minute. For tandem mass spectrometry detection, the data-dependent acquisition (DDA) mode was used. MS/MS spectra was acquired with a scan resolution of 60,000 full width at half maximum and a mass-to-charge ratio of 350–2,000 m/z. Peptides were fragmented using higher-energy collisional dissociation at 28% normalized collision energy.

### 2.5 Proteomic data analysis

The raw data were processed with MaxQuant (version 1.5.8.3) using the settings for the human protein database UniProt. Reverse and potential contaminant proteins were removed. Carbamidomethyl (C) was set as a fixed modification, whereas oxidation (M) and acetyl (protein N-term) were variable modifications. The minimal peptide length was set to seven amino acids, and two missed cleavages were set as the maximum peptide length. The results were filtered at a 0.01 false discovery rate (FDR) for peptides and 0.05 FDR for proteins. The proteins were quantified according to label-free quantitation(LFQ) intensity. Only proteins with 100% missing values were excluded for differential analysis. Missing values were imputed with half the minimum LFQ value of all proteins. Non-log transformed LFQ values of the proteins were used for analysis.

### 2.6 Multiple reaction monitoring analysis

The candidate biomarkers were verified by MRM analysis. In brief, a peptide assay library was generated for these proteins using LC–MS/MS and Skyline analysis software (version 19.1). Optimal peptides selected manually by Skyline were analyzed through scheduled MRM analysis. MRM data were acquired on a SCIEX QTRAP 6500 coupled to an Ekspert nanoLC 425 liquid chromatography system using MRM scanning in positive mode (Framingham, MA). The raw data were analyzed with Skyline software, and the peak areas were subjected to quantitative analysis. Both.wiff and.wiff.scan raw data were imported into Skyline software, and then the retention time of the peptide peaks was corrected, and the peak area of each peptide was analyzed. The predictor Mz (m/z), best retention time and total area values of each peptide were saved, where the total area value was used for quantification. Finally, the total area values of peptides belonging to the same protein were added up as the quantitative result of the protein.

### 2.7 Bioinformatics analysis

Reactome pathway analysis was performed using the DAVID web tool (http://david.ncifcrf.gov/). Up- and downregulated differentially expressed proteins as separate input lists were analyzed. Only significant pathways with FDR < 0.05 were considered for analysis. Protein–protein network analysis was conducted using the Metascape web tool (metascape.org). All identified proteins with log2 transformed LFQ values were used for principal component analysis (PCA) via a built-in function named *`prcomp`* in the R language program. The gene set enrichment analysis (GSEA) method (http://www.gsea-msigdb.org/) was used to identify significantly enriched or depleted groups of proteins.

### 2.8 Analysis of the NB database

Marker genes for tumor cells, chromaffin cells, and sympathoblasts were downloaded directly from two published studies (30, 31). scRNA-seq data from NB tissues (GSE137804) were downloaded and reanalyzed to determine which cell types mainly expressed the sEVs protein biomarkers.

The Sequencing Quality Control Consortium (SEQC) cohort, which included 498 NB samples with transcriptomic data (32), was used to analyze the associations between sEVs biomarkers and patient outcomes. This analysis can be implemented on the R2: Genomics Analysis and Visualization Platform (http://r2.amc.nl). Briefly, the SEQC data set was selected on the R2 platform, and then the DEPs were subjected to Cox proportional hazards regression analysis with the aim of identifying independent factors that have a significant impact on patient overall survival. As a result, proteins that were significantly related to survival rate were first filtered based on p<0.01. Subsequently, among the upregulated DEPs in NB, those with a significant negative correlation with survival rate were selected, while the downregulated DEPs that showed a significant positive correlation with survival rate were selected. Two-tailed Student’s t test was used to compare the gene expression differences between high-risk and low-risk NB patients.

### 2.9 Logistic regression model

The dataset of the validation cohort was divided into two parts: a training set (50%) and a testing set (50%). The training set was used to train the logistic regression model, while the testing set was used to evaluate its performance. Tenfold cross-validation was used to assess the performance of the model in the training set. Receiver operating characteristic (ROC) curves were constructed using the R package “pROC”, and the area under the curve (AUC) was calculated to evaluate the performance of the trained model in the testing set.

### 2.10 Statistical analysis

The statistical significance of differences in the sEVs proteome between different groups was determined with an unpaired two-tailed Student’s t test. Categorical and continuous variables were summarized using percentages and medians and interquartile ranges, respectively. The statistical significance of differences in clinical categorical variables was assessed using Fisher’s exact test, whereas the Mann−Whitney U test was used for nonparametric comparisons between different groups. A p value of < 0.05 was considered to indicate statistical significance.

## 3 Results

### 3.1 Proteomic profile of serum sEVs

**Figure 1:**
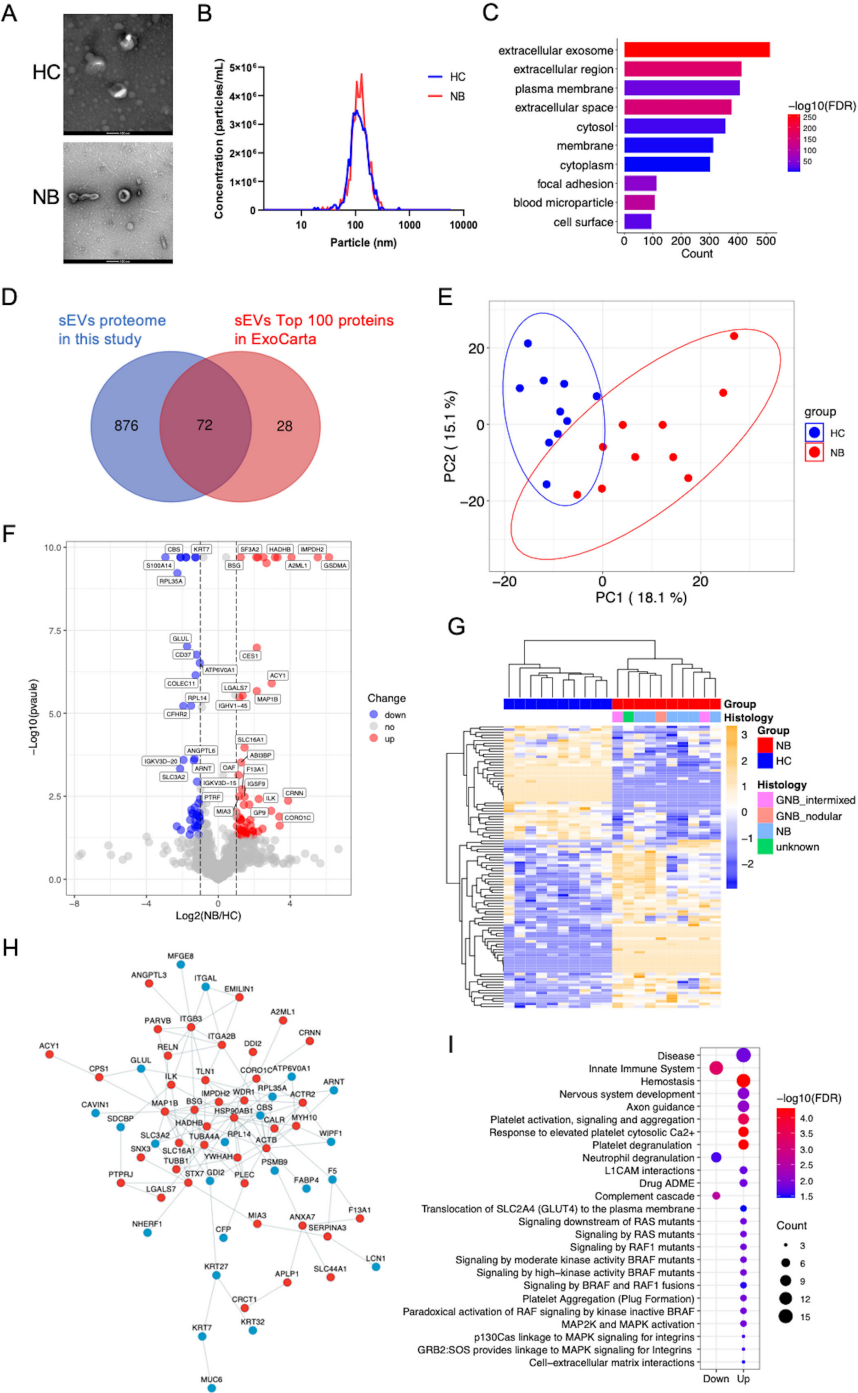
Characterization and proteomic profile of serum sEVs. **(A)** Representative TEM images of sEVs. Bars, 100 nm. The sEVs in the field of view show a typical cup-shaped structure. **(B)** The size distribution of the particles. The diameter of most sEVs particles is distributed in the range of about 100nm. **(C)** Bar chart showing the distribution of the GO cellular component terms. The top ten overrepresented categories with p < 0.05 are shown. **(D)** Venn diagram displaying the number and overlap of proteins identified from serum sEVs in the present study and exosomal top 100 proteins in the Exocarta database. **(E)** PCA of the twenty samples based on all proteins identified. **(F)** Volcano plot indicating the log2-fold change in protein abundance in NB patients relative to HC. Red, blue and gray dots represent significantly upregulated, downregulated and unchanged proteins respectively. The identification of proteins with significant expression changes is based on the following principles: logFC >1 or < -1 and p < 0.05. **(G)** Heatmap of 104 DEPs. **(H)** Protein–protein interaction network of the DEPs was visualized using Cytoscape. The red and blue dots show proteins whose expression significantly increased and decreased, respectively. **(I)** Bubble plot of reactome pathway enrichment analysis of the upregulated (Up) and downregulated (Down) DEPs using DAVID. The colors of the nodes are shown from red to blue in descending order for the –log10 FDR. The sizes of the nodes are shown from small to large in ascending order for the number of proteins. NB, neuroblastoma; HC, healthy control; GNB intmixed, ganglioneuroblastoma intermixed; GNB nodular, ganglioneuroblastoma nodular.

In the discovery phase, we isolated sEVs from the serum of 10 healthy individuals and 10 NB patients. The sEVs preparations presented a typical cup-shaped morphology (**Figure 1A**). The diameter of most of the particles ranged between 50 and 150 nm (**Figure 1B**), coinciding with the size range of sEVs defined by the International Society for Extracellular Vesicles (33). Two sEVs markers, Hsp70 and CD9, were detected in the sEVs separated from both NB patients and HC (**Supplementary Figure 1**). A total of 948 sEVs proteins with ≥1 unique peptide in all the samples were identified (**Supplementary Tables 2**, **3**). Most of the proteins identified were classified as components of extracellular exosomes, extracellular space and region, plasma membrane or cytosol (**Figure 1C**). Among the 100 most frequently identified sEVs proteins from the ExoCarta database, 72 were identified in the serum sEVs proteome in the present study (**Figure 1D**). These results indicated that the sEVs were collected with high purity.

We performed PCA of all the sEVs proteins identified, which clearly separated the samples into two groups (**Figure 1E**). We identified 104 differentially expressed proteins (DEPs) (**Supplementary Table 4**), with 62 upregulated and 42 downregulated proteins in the NB patients compared with the HC (**Figure 1F**). Unbiased hierarchical clustering analysis of these DEPs revealed that they correctly clustered together the samples from patients with NB (**Figure 1G**). These DEPs can form a tight interaction network (**Figure 1H**). Reactome pathway analysis illustrated that the upregulated proteins in NB were principally enriched in pathways related to neural development, platelet activity, RAF mutants mediated signaling and MAPK pathways, while the downregulated proteins were enriched in innate immune system, complement and neutrophil degranulation related pathways (**Figure 1I**, **Supplementary Table 5**), consistent with the high relevance of these pathways to NB, especially as activation of the RAS-MAPK pathway has been found to be associated with more severe phenotypes in NB (34).

### 3.2 Analysis of the associations between DEPs and NB prognosis

**Figure 2:**
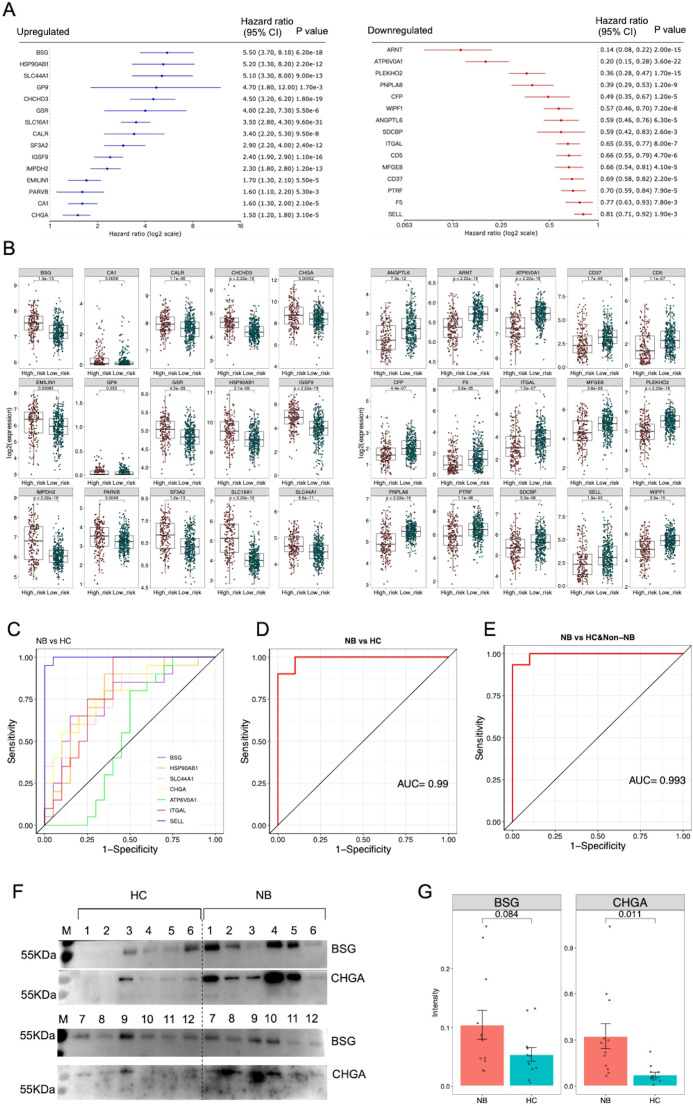
Analysis of potential sEVs protein biomarkers. **(A)** The genes encoding DEPs were subjected to Cox proportional hazards regression analysis using the NB SEQC dataset containing transcriptomic data from 498 samples on the R2: Genomics Analysis and Visualization Platform (http://r2.amc.nl). The 15 upregulated proteins were significantly negatively correlated with survival rate, while the 15 downregulated proteins were significantly positively correlated with survival rate (p<0.01). **(B)** Boxplots showing the expression of prognosis-related genes in high-risk NB (n=176) compared with low-risk NB (n=322). Two-tailed Student’s t test was used to compare the gene expression differences between high-risk and low-risk NB patients. **(C)** ROC curve analysis of 7 protein biomarkers for distinguishing NB patients from HC. **(D)** ROC curve analysis of a logistic regression model based on 7 protein biomarkers for distinguishing NB patients from HC. **(E)** ROC curve analysis of the logistic regression model for distinguishing NB patients from HC and non-NB patients. **(F)** The results of western blot analysis of BSG and CHGA proteins. A total of 12 healthy individuals and 12 patients’ samples were tested. **(G)** Statistical analysis of semi-quantitative results of bands analyzed by Image J software.

To highlight the potential of serum sEVs proteins as NB biomarkers, we analyzed the relationships between genes encoding DEPs and NB prognosis in the NB SEQC dataset containing transcriptomic data from 498 samples (32). Cox regression analysis showed that a total of 15 upregulated proteins—BSG, HSP90AB1, SLC44A1, GP9, CHCHD3, GSR, SLC16A1, CALR, SF3A2, IGSF9, IMPDH2, EMLIN1, PARVB, CA, and CHLI—and 15 downregulated proteins—ARNT, ATP6V0A1, PLEKHO2, PNPLAB, CFP, WIPF1, ANGPTL6, SDCBP, ITGAL, CD5, MFGE8, CD37, PTRF, F5 and SELL—were independently associated with the survival of NB patients (**Figure 2A**). Consistently, genes encoding upregulated proteins were expressed at higher levels in high-risk NB than in low-risk NB, while genes encoding downregulated proteins showed the opposite pattern (**Figure 2B**). Collectively, these findings imply that the serum sEVs have potential as biomarkers for evaluating clinical outcomes in patients with NB.

### 3.3 Validation of the serum sEVs protein biomarkers by MRM analysis

We selected 45 sEVs proteins identified in the discovery phase for evaluation and quantification through MRM analysis in an independent cohort of 20 NB patients, 20 HC and 10 non-NB cancer patients. In particular, among these validated proteins, we specifically selected 13 membrane proteins that were significantly upregulated in neuroblastoma, including PGLYRP2, GP9, SLC44A1, STX7, PARVB, PTPRJ, SLC16A1, ITGB3, BSG, SLC43A3, ITGA2B, MIA3 and APLP1, as well as a typical neuroblastoma marker CHGA, while the remaining 32 proteins were selected randomly (**Supplementary Table 6**). As a result, a total of 39 peptides representing 29 proteins were successfully detected by MRM (**Supplementary Tables 7**, **8**). The differential trends in the expression levels of 26 proteins were consistent with the quantitative results of the label-free proteomic analysis (**Table 1**). Remarkably, these proteins demonstrated high ability for distinguishing NB patients from HC (**Figure 2C**) (**Supplementary Table 9**).

**Table 1:** List of proteins successfully detected by MRM analysis.

Gene Symbol	Discovery	Validation			
	NB vs HC	NB vs HC		NB vs non-NB tumors	
	LogFC	LogFC	Pvalue	LogFC	Pvalue
**A2ML1**	**4.072**	**1.756**	**3.95E-13**	**1.602**	**2.23E-08**
**PGLYRP2**	**3.416**	**1.555**	**3.98E-09**	**2.000**	**9.62E-07**
**ACY1**	**2.970**	**1.956**	**2.44E-12**	**1.904**	**4.32E-08**
F9	2.462	2.410	0.066	0.384	0.733
**ANGPTL2**	**2.206**	**1.021**	**3.46E-05**	**1.076**	**0.0008**
**ANGPTL3**	**1.930**	**2.851**	**2.61E-06**	**1.574**	**0.010**
**HSP90AB1**	**1.923**	**1.265**	**0.041**	0.605	0.395
**SLC44A1**	**1.916**	**1.175**	**0.001**	**1.372**	**0.006**
**CHGA**	**1.804**	**2.419**	**0.014**	1.337	0.197
**STX7**	**1.573**	**2.063**	**0.002**	**1.784**	**0.042**
**PTPRJ**	**1.491**	**0.955**	**2.14E-05**	**0.918**	**0.003**
MYH10	1.452	0.743	0.066	0.724	0.182
LGALS7	1.354	1.477	0.116	2.921	0.123
**GNPTG**	**1.255**	**2.782**	**1.71E-08**	**1.303**	**0.011**
**BSG**	**1.247**	**0.903**	**0.011**	0.547	0.188
**ITGA2B**	**1.177**	**1.178**	**3.91E-06**	0.502	0.061
**MIA3**	**1.161**	**2.202**	**1.61E-06**	**1.879**	**0.0007**
**APLP1**	**1.138**	**1.624**	**8.44E-07**	**1.056**	**0.003**
**HLA-B**	**0.968**	**1.177**	**0.0005**	**1.387**	**0.003**
**PIGR**	**-0.735**	**-0.875**	**0.0005**	-0.446	0.068
DPP4	-0.832	-0.147	0.754	-0.868	0.097
SEPP1	-0.851	-0.402	0.236	-0.828	0.036
FGL2	-0.881	0.557	0.219	0.038	0.930
ATP6V0A1	-1.030	-0.277	0.354	-0.279	0.453
**SELL**	**-1.092**	**-1.681**	**5.84E-13**	**-1.816**	**6.60E-12**
**CHL1**	**-1.153**	**-1.556**	**2.76E-05**	**-1.777**	**4.06E-06**
PNPLA8	-1.185	-0.397	0.157	0.076	0.818
**ITGAL**	**-2.083**	**-0.791**	**0.0003**	**-0.529**	**0.011**
SLC3A2	-2.124	0.876	0.012	1.149	0.008

The proteins marked in bold indicate significant differences in expression.

FC, fold change; LogFC, log2 (FC).

Subsequently, we selected 7 proteins including BSG, HSP90AB1, SLC44A1, CHGA, ATP6V0A1, ITGAL and SELL that were significantly differentially expressed in the discovery and validation phases and were significantly related to patient prognosis in the NB SEQC cohort to construct a logistic regression model. ROC analysis revealed that this model had high discriminatory power for distinguishing NB patients from HC or a combined population of HC and non-NB patients (**Figures 2D, E**). Furthermore, we randomly selected and matched 12 NB patients and 12 healthy individuals with available samples (**Supplementary Table 10**), and detected five proteins (BSG, HSP90AB1, SLC44A1, CHGA, and ITGAL) in sEVs from these participants using western blot analysis. The results showed that the expression levels of CHGA and BSG proteins in patients were higher than that in healthy individuals, although not significantly for BSG (**Figures 2F, G**). Unfortunately, HSP90AB1, SLC44A1, and ITGAL were not detected (**Supplementary Figure 2**), perhaps due to the low sensitivity of western blot for detecting these proteins, suggesting the need for more sensitive strategies for detecting sEVs proteins. Collectively, these data suggested that these sEVs proteins, if validated in a large cohort, may serve as potential biomarkers for the diagnosis of NB.

### 3.4 Proteomic profile of serum sEVs reflects the molecular characteristics of neuroblastoma tissue

**Figure 3:**
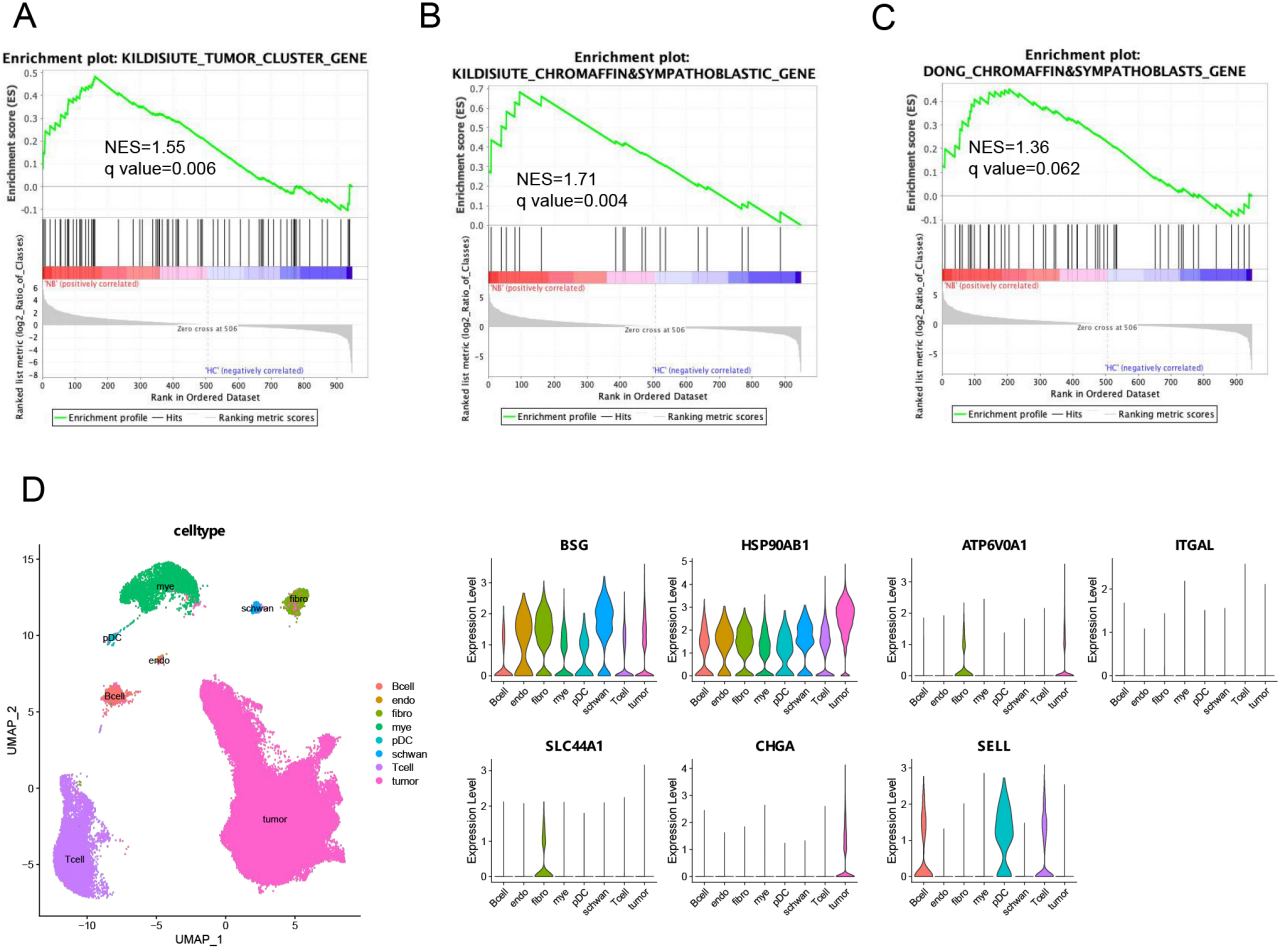
Integrated analysis of the serum sEVs proteome and NB tissue scRNA-seq data. **(A–C)** GSEA of three gene sets from published neuroblastoma scRNA-seq databases, including genes highly expressed by tumor cells or chromaffin and sympathoblastic cells, in the serum sEVs proteome. The leading edges (most significant genes) are shown as vertical bars accumulated below the peak of the green enrichment score plot, indicating the upregulated genes identified by GSEA characterized by the highest normalized enrichment score (NES). **(D)** UMAP plot and cell type proportions in NB tumors (GSE137804). Violin plots show that biomarker rexpressing-positive cells and relative biomarker expression within the cell types of NB tumors. endo, endothelial cells; fibro, fibroblasts; mye, myeloid cells; pDC, plasmacytoid dendritic cells.

We speculated that the serum sEVs in NB patients may be partly derived from tumor cells and that the molecular profile of sEVs may also be partially consistent with that of NB tumor cells. To this end, we used the marker genes of tumor cells from a published NB tissue scRNA-seq database (30) as the reference gene set for GSEA. These tumor cell markers were positively correlated with the protein signature of sEVs in the NB patients compared with those in the HC (**Figure 3A**). In addition, malignant NB cells have molecular characteristics similar to those of chromaffin cells and sympathoblasts. The expression of the marker genes of chromaffin cells and sympathoblasts was also positively correlated with the protein signature of NB sEVs (**Figures 3B, C**). Taken together, the proteomic profile of serum sEVs partially reflects the transcriptional signatures of malignant cells in NB patients.

Furthermore, to reveal which cell types in the tumor microenvironment may release sEVs carrying potential biomarkers, we investigated the expression of seven candidate serum sEVs protein biomarkers in cell populations within human NB tumors (**Figure 3D**). According to the analysis of the single-cell transcriptome of the GSE137804 dataset, the cells with the highest percentages of CHGA and SLC44A1 transcripts were malignant NB cells and fibroblasts, respectively, whereas ATP6V0A1 was expressed by both malignant cells and fibroblasts, and SELL-expressing cells were the main immune cell subpopulation, which included B cells, T cells and plasmacytoid dendritic cells (pDCs). Moreover, the expression of BSG and HSP90AB1 was more widespread than that of the other genes and was detected in all the main cell populations. These findings imply that sEVs loaded with these proteins may play a crucial role in mediating communication between different cells in the microenvironment, and the underlying mechanisms should be further explored.

## 4 Discussion

Herein, we demonstrated that serum sEVs contain protein biomarkers for the diagnosis of NB. This has significant clinical relevance, as current NB diagnostic markers often require confirmation through certain invasive methods, increasing the complexity and cost of the diagnostic process. Based on the fact that sEVs are easy to collect from clinical samples and their cargos are not easily degraded, as well as their molecular profile has the ability to reflect the genetic and molecular characteristics of tumor tissue, the potential of sEVs as biomarkers has attracted increasing attention (12). Herein, we displayed that expression levels of potential sEVs biomarkers were also significantly associated with patient prognosis according to transcriptomic data from a public large-scale cohort (32), and revealed that these potential biomarkers can be expressed by malignant cells or other cells of the tumor microenvironment by analyzing a public scRNA-seq dataset of NB tissues (30). These findings highlight the potential of detecting serum sEVs proteins as a novel strategy to improve NB detection and management.

What needs attention is that the isolation of high-purity sEVs should be the first concern in sEVs research. There are different separation methods available, such as ultracentrifugation, density gradient centrifugation, size exclusion chromatography (SEC), and immunoaffinity ultrafiltration, that are chosen to separate sEVs depending on the type and volume of the sample (35). In the present study, qEV columns were used to isolate sEVs from serum. qEV columns use principles of SEC to collect sEVs. Advantages of SEC include the efficient separation of sEVs from soluble proteins and allowing the recovery of pure vesicles with intact and biologically activity (36). Similar to previous studies, we analyzed fractions 7–9 separated by qEV columns mainly containing sEVs, and then confirmed the shape and size of sEVs using NTA and TEM analysis, ensuring that sEVs were the primary objects analyzed in this study.

Tracing the expression and distribution of biomarkers carried by circulating sEVs in tumor tissues facilitates understanding the associations between these sEVs and the degree of tumor progression and the underlying biological significance. In this study, we showed that the tissue transcription levels of regulated sEVs proteins in NB patients compared with healthy subjects were significantly correlated with the risk grade and prognosis of NB patients, suggesting that these sEVs proteins are closely associated with NB. In particular, seven proteins including BSG, HSP90AB1, SLC44A1, CHGA, ATP6V0A1, ITGAL and SELL had a high ability to distinguish NB patients from other individuals. Integrated analysis of scRNA-seq data revealed that these seven sEVs-derived biomarkers originated from different cell populations in tumor tissues. Apparently, CHGA, a typical NB-related protein, was exclusively expressed in tumor cells. Elevated levels of CHGA in the blood or urine have been shown to indicate the presence of NB or the progression of the disease (37). Many studies have shown that measuring CHGA can aid in assessing the severity of NB and guiding treatment decisions (38). Whether monitoring CHGA carried by sEVs has more advantages than previous methods of detecting CHGA needs further verification. Three proteins, BSG, HSP90AB1, and SLC44A1, have been shown to be involved in the metastasis, proliferation, and progression of NB. BSG, also known as CD147, has been identified in NB cell line-derived sEVs (39), and targeting BSG in NB has been explored as a potential therapeutic strategy (40). HSP90AB1 is associated with protein folding, stabilization, and degradation. Studies have shown that HSP90AB1 inhibition can lead to the degradation of client proteins that are critical for NB cell survival and proliferation (41). The two genes BSG and HSP90AB1are widely expressed in different cell populations in tumor tissues, suggesting that sEVs carrying these two proteins may have complex functions in regulating the tumor microenvironment. Altered SLC44A1 expression could impact choline transport and metabolism, which may have implications for NB development and progression (42). We found that SLC44A1 is specifically expressed in fibroblasts of NB tissues, indicating that sEVs containing this protein may mediate communication between fibroblasts and tumor cells or other cells in the NB tumor microenvironment. SELL, also known as L-selectin or CD62L, is a cell adhesion molecule that is primarily expressed on naive and central memory T cells and is involved in lymphocyte trafficking and homing (43). ITGAL, also known as CD11a or LFA-1, is a critical leukocyte adhesion molecule in leukocyte arrest and immunological synapse formation (44). The expression levels of SELL and ITGAL in circulating sEVs of NB patients may indicate the activation or suppression status of T cells or other immune cells in tumor tissues. A recent study demonstrated that ATP6V0A1, a subunit of the V-ATPase (vacuolar ATPase) enzyme, facilitates cholesterol uptake in colorectal cancer cells to trigger immunosuppressive signals to inactivate memory CD8^+^ T cells (45). We also found that ATP6V0A1 is highly expressed in tumor cells and fibroblasts of NB tissues. However, the role of ATP6V0A1 and ATP6V0A1-carrying sEVs in NB need to be further studied. Overall, understanding the functions and interactions of these proteins with sEVs may reveal new avenues for cancer diagnosis and treatment.

Although the current study has shed lights on the potential of sEVs as biomarkers for diagnosis of NB, several aspects can be improved including increasing the sample size to improve the statistical power and allow more conclusive statements to be made on the associations between sEVs proteins and NB. In addition, biomarkers for patients with different histological subtypes need to be determined, especially for distinguishing high-risk and low-risk patients. In the future, validation studies involving larger patient cohorts and standardized measurement techniques are necessary to establish the reliability and effectiveness of serum sEVs protein biomarkers as diagnostic or prognostic tools for the purpose of enhancing early diagnosis, improving treatment strategies, and ultimately positively impacting patient outcomes.

## Data Availability

The data presented in the study are deposited in the ProteomeXchange repository, accession number PXD048511.

## Supplementary material

Supplementary Figure 1 Western blot analysis of sEVs. Left: western blot analysis of sEVs using CD9 antibody; Right: western blot analysis of sEVs using HSP70 antibody. Protein markers were showed. HC: healthy controls; NB: neuroblastoma.

Supplementary Figure 2 Western blot analysis of sEVs samples isolated from 12 HC and 12 NB patients. BSG and CHGA were detected both from HC and NB samples, whereas HSP90AB1, ITGAL, and SLC44A1were not detected in both samples.

Supplementary Table 1 Clinical characteristics of the participants. Histologic category of neuroblastoma (NB) is based on International Neuroblastoma Pathology Classification (INPC)(1). One female NB patient could not be classified because her primary tumor tissue was not obtained.

Supplementary Table 2 List of identified peptides by LC-MS/MS. LC-MS/MS analysis of sEVs from 10 HC and 10 NB samples in the discovery phase. Identified peptides are shown.

Supplementary Table 3 The peak intensities of 948 proteins with ≥1 unique peptide. Proteins matched to peptides identified by LC-MS/MS in the discovery phase are shown. The peak intensities of 948 proteins with ≥1 unique peptide are displayed.

Supplementary Table 4 List of 104 DEPs. In the discovery phase, proteins that were significantly differentially expressed between the NB and HC groups are displayed.

Supplementary Table 5 Reactome pathway analysis of DEPs using DAVID. In the discovery phase, pathways that were significantly enriched for upregulated or downregulated proteins in NB patients compared to healthy controls are shown.

Supplementary Table 6 List of 45 proteins selected for MRM validation. The 45 proteins identified in the discovery phase were selected for MRM detection in the validation phase. The differences of these proteins between the NB and HC groups in the discovery phase are shown.

Supplementary Table 7 Peak areas of 39 peptides analyzed by MRM analysis. In the validation phase, sEVs from 20 HC, 20 NB, and 10 non-NB samples were detected by MRM. Among the peptides of the selected 45 proteins, 39 peptides were successfully detected by MRM, and their peak areas are shown.

Supplementary Table 8 Peak areas of 29 proteins analyzed by MRM analysis. Proteins matched to 39 peptides identified by MRM in the validation phase are shown. The peak areas of 29 proteins are displayed.

Supplementary Table 9 ROC analysis of sEVs protein biomarkers for distinguishing NB from HC. In the validation phase, for each sEVs protein biomarker, the cutoff value, sensitivity, specificity, and area under the ROC curve (AUC) with the corresponding 95% confidence interval (CI) are shown. The NB patients were differentiated from the HC by using a dichotomous variable. The cutoff values were determined based on the best performing Youden index.

Supplementary Table 10 Clinical characteristics of the participants included in the Western blot validation cohort. Histologic category of neuroblastoma (NB) is based on International Neuroblastoma Pathology Classification (INPC) (46).
