# Supplementary figures and images for: Proteomic analysis of serum small extracellular vesicles identifies diagnostic biomarkers for neuroblastoma
Source: Front Oncol. 2024 Aug 20;14:1367159. doi: 10.3389/fonc.2024.1367159 (PMC11368728; doi:10.3389/fonc.2024.1367159)

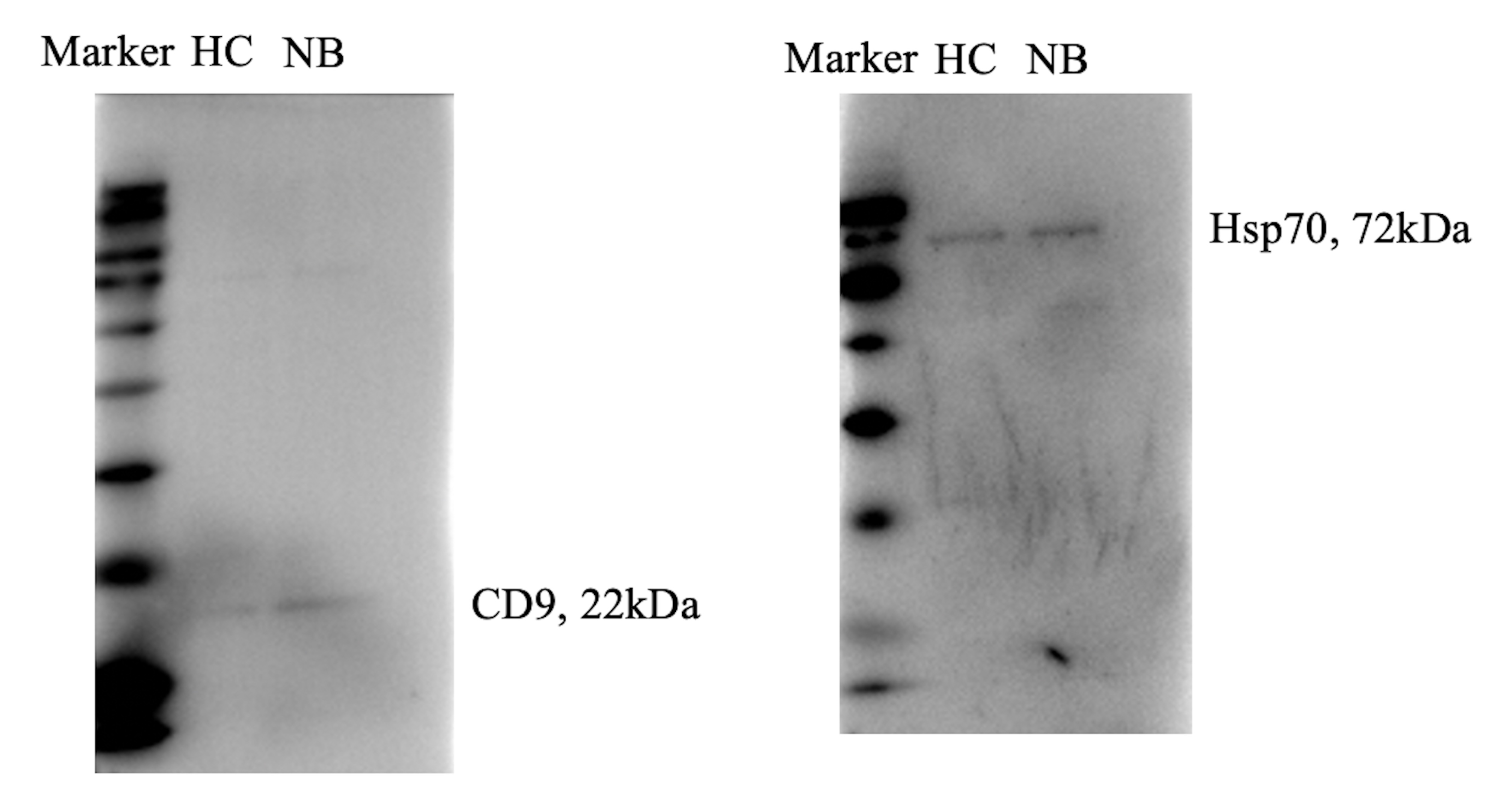

Supplement: Supplementary Figure 1 — Western blot analysis of sEVs. Left: western blot analysis of sEVs using CD9 antibody; Right: western blot analysis of sEVs using HSP70 antibody. Protein markers were showed. HC: healthy controls; NB: neuroblastoma. [file Image1.jpeg]

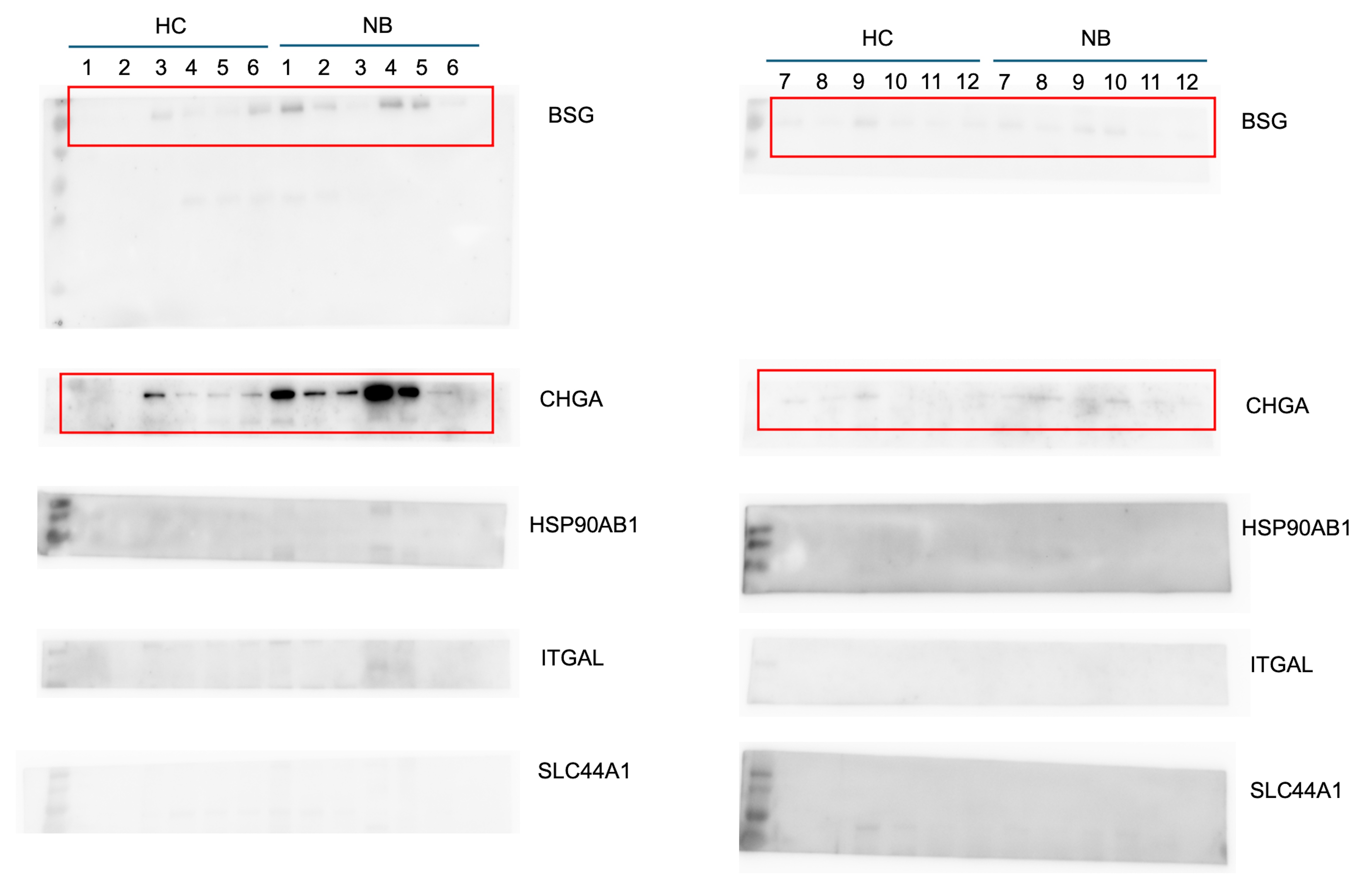

Supplement: Supplementary Figure 2 — Western blot analysis of sEVs samples isolated from 12 HC and 12 NB patients. BSG and CHGA were detected both from HC and NB samples, whereas HSP90AB1, ITGAL, and SLC44A1were not detected in both samples. [file Image2.jpeg]
